# Receptor-Activator of Nuclear KappaB Ligand Expression as a New Therapeutic Target in Primary Bone Tumors

**DOI:** 10.1371/journal.pone.0154680

**Published:** 2016-05-10

**Authors:** Tetsuro Yamagishi, Hiroyuki Kawashima, Akira Ogose, Takashi Ariizumi, Taro Sasaki, Hiroshi Hatano, Tetsuo Hotta, Naoto Endo

**Affiliations:** 1 Division of Orthopedic Surgery, Niigata University Graduate School of Medical and Dental Sciences, Niigata, Japan; 2 Department of Orthopedic Surgery, Uonuma Kikan Hospital, Minamiuonuma, Niigata, Japan; 3 Department of Orthopedic Surgery, Niigata Cancer Center Hospital, Niigata, Japan; Charles P. Darby Children's Research Institute, 173 Ashley Avenue, Charleston, SC 29425, USA, UNITED STATES

## Abstract

The receptor-activator of nuclear kappaB ligand (RANKL) signaling pathway plays an important role in the regulation of bone growth and mediates the formation and activation of osteoclasts. Osteoclasts are involved in significant bone resorption and destruction. Denosumab is a fully human monoclonal antibody against RANKL that specifically inhibits osteoclast differentiation and bone resorption. It has been approved for use for multiple myeloma and bone metastases, as well as for giant cell tumor of bone. However, there is no previous report quantitatively, comparing RANKL expression in histologically varied bone tumors. Therefore, we analyzed the mRNA level of various bone tumors and investigated the possibility of these tumors as a new therapeutic target for denosumab. We examined RANKL mRNA expression in 135 clinical specimens of primary and metastatic bone tumors using real-time PCR. The relative quantification of mRNA expression levels was performed via normalization with RPMI8226, a human multiple myeloma cell line that is recognized to express RANKL. Of 135 cases, 64 were also evaluated for RANKL expression by using immunohistochemistry. Among all of the tumors investigated, RANKL expression and the RANKL/osteoprotegerin ratio were highest in giant cell tumor of bone. High RANKL mRNA expression was observed in cases of aneurysmal bone cyst, fibrous dysplasia, osteosarcoma, chondrosarcoma, and enchondroma, as compared to cases of multiple myeloma and bone lesions from metastatic carcinoma. RANKL-positive stromal cells were detected in six cases: five cases of GCTB and one case of fibrous dysplasia. The current study findings indicate that some primary bone tumors present new therapeutic targets for denosumab, particularly those tumors expressing RANKL and those involving bone resorption by osteoclasts.

## Introduction

The receptor-activator of nuclear kappa B ligand (RANKL) signaling pathway plays an important role in the regulation of bone growth and turnover. RANKL is an essential regulator of osteoclastogenesis, and it is expressed on the surface of osteoblasts or stromal cells [1]. Moreover, it mediates the formation and activation of multinucleated osteoclasts from RANK-positive mononuclear preosteoclasts and macrophages [1], and osteoclasts cause significant bone resorption and destruction in some pathological bone lesions. Osteoprotegerin (OPG) is a soluble member of the tumor necrosis factor receptor superfamily, acts as a decoy receptor for RANKL [2,3], and inhibits the stimulation of osteoclast differentiation in conjunction with RANKL [2,3].

Denosumab is a fully human monoclonal antibody against RANKL that specifically inhibits osteoclast differentiation and bone resorption by preventing the RANKL-mediated formation and activation of osteoclasts [4,5,6]. It has been shown to suppress bone destruction in patients with osteolytic bone disease in multiple myeloma, bone metastases from solid cancer, and giant cell tumor of bone (GCTB) [7,8]. In 2010, Thomas et al. reported that denosumab had an effect GCTB, as determined histologically and radiologically [9]; in addition, Chakarun et al. reported that preoperative treatment with denosumab made surgical resection easier [10]. Denosumab is approved for the treatment of these tumors, which express RANKL and involve osteoclast activation [4].

RANKL expression and the therapeutic efficacy of denosumab have recently been reported in various other giant cell-rich neoplasms that cause bone resorption [11,12,13]. However, to our knowledge, no previous report has quantitatively compared RANKL mRNA expression in histologically varied primary bone tumors.

Hence, we aimed to analyze RANKL expression by real-time PCR and immunohistochemistry in various primary bone tumors and determine the possibility as new therapeutic targets with denosumab, based on the idea that these findings would help to identify RANKL-expressing bone tumors for which denosumab may be an effective treatment.

## Materials and Methods

### Specimens

Clinical specimens were obtained from 135 total patients with bone tumors, treated in our institutes between 2007 and 2014. Cases included 63 male (46.7%) and 72 female (53.3%) patients (Table 1). The histological types included GCTB (n = 18), chondrosarcoma (n = 17), osteosarcoma (n = 16), osteochondroma (n = 12), and other various types. The specimens were obtained by using core-needle biopsy, incisional biopsy, or surgical resection. The diagnosis was confirmed histopathologically according to the World Health Organization classification [14]. Two experienced pathologists independently diagnosed each case. In cases that were difficult to diagnose, the pathologists reviewed and discussed the cases carefully and reached a consensus. Written informed consent was obtained from all patients for participation in this study, and this study was approved by the Ethics Committee of School of Medicine, Niigata University.

**Table 1:** List of histological types and numbers of clinical specimens.

Histology	Cases (n = 135)	Sex	
		M (n = 63)	F (n = 72)
**Giant cell tumor of bone**	18	14	4
**Chondrosarcoma**	17	6	11
**Osteosarcoma**	16	7	9
**Osteochondroma**	12	9	3
**Fibrous dysplasia**	10	4	6
**Enchondroma**	9	3	6
**Ewing’ sarcoma**	8	1	7
**Aneurysmal bone cyst**	8	3	5
**Chordoma**	4	1	3
**Solitary bone cyst**	3	0	3
**Osteoid osteoma**	2	1	1
**Osteofibrous dysplasia**	2	1	1
**Multiple myeloma**	3	1	2
**Osteochondromatosis**	1	1	0
**Clear cell chondrosarcoma**	1	0	1
**Ossifying fibroma**	1	1	0
**Non-ossifying fibroma**	1	1	0
**Leiomyosarcoma**	2	0	2
**Chondroblastoma**	1	1	0
**Osteoma**	1	0	1
**Bone metastasis**	15	8	7

### Quantitative real-time PCR

First, total RNA was extracted from frozen samples using ISOGEN (Nippon Gene, Tokyo, Japan). The yield and purity of RNA were determined based on spectrophotometric measurements of the ratio of UV absorbance at 260 and 280 nm. First strand cDNA was synthesized from the total RNA by using PrimeScript RT Reagent Kit (TaKaRa Bio, Shiga, Japan). Quantitative real-time PCR was performed using SYBR Premix EX Taq II (Tli RNaseH Plus; TaKaRa, Shiga, Japan), and the results were analyzed using the Thermal Cycler Dice Real Time System TP800 (TaKaRa, Shiga, Japan). The primer sequences used were as follows: primer pairs used for human RANKL, 5′-GCCTTTCAAGGAGCTGTGCAA-3′, (forward) and 5′-ATCTAACCATGAGCCATCCACCAT-3′ (reverse); RANK, 5′-GCCATCATCTTTGGCGTTTG-3′ (forward) and 5′-CAAAGTTTGCCGTGTGTGTACTG-3′ (reverse); OPG 5′-CAATTTGCCTGGCACCAAAG-3′ (forward) and 5′-AGGTGAGGTTAGCATGTCCAATGT-3′ (reverse); GAPDH, 5′- GCACCGTCAAGGCTGAGAAC-3′ (forward) and 5′-TGGTGAAGACGCCAGTGGA-3′ (reverse).

The gene copy numbers for RANKL, RANK and OPG were calculated using a standard curve that was constructed using the Saos2 cell line. Moreover, quantitative mRNA expression levels were calculated by normalizing against RPMI 8226, a human multiple myeloma cell line that expresses RANKL.

We compared the median of the relative expression level in all histological types of tumors by using a box plot for expression of RANKL, RANK, and OPG. For histological types in which only one case was sampled, we used only a dot plot.

### Immunohistochemistry

RANKL expression was valued by using immunohistochemistry. Among 135 patients, 66 specimens were evaluable for RANKL expression. Histological types determined in these expreriments are shown in Table 2. Formalin-fixed, paraffin-embedded (FFPE) tissue sections (4-um) had the paraffin removed and were hydrated. The sections were heated with an autoclave (121°C for 20 minutes) in histofine antigen retrieval buffer, pH 9 (Nichirei Bioscience, Tokyo, Japan), and blocked against endogenous peroxidase activity and staining reagent. Tissue sections were incubated overnight at 4°C with rabbit anti-human RANKL antibody (ab9957; Abcam, Cambridge, UK). Primary antibodies were visualized by using the histofine Simple Stain MAX-PO (MULTI) kit (Nichirei Bioscience) and 3, 3’-diaminobenzidine (Simple Stain DAB, Nichirei Bioscience. The sections were counterstained in Mayer’s hematoxylin.

**Table 2:** List of histological types and numbers assessed by immunohistochemistry.

Histology	cases (n = 66)
**Giant cell tumor**	11
**Chondrosarcoma**	13
**Osteosarcoma**	5
**Fibrous dysplasia**	9
**Ewing’ sarcoma**	7
**Aneurysmal bone cyst**	8
**Chordoma**	2
**Multiple myeloma**	1
**Leiomyosarcoma**	2
**Bone metastasis**	8

### Statistical analysis

Statistical analysis was conducted with the Statistical Package for the Social Sciences (SPSS Inc. Chicago, Illi-nois, USA) version 21.0. We performed the statistical analysis for the 15 histological types for which there were more than two cases. As the data were not normally distributed as determined by using Shapiro-Wilk test, they were analyzed with a post-hoc multiple comparison. The significance level was set at P < 0.05.

## Results

### RANKL expression

**Fig 1:**
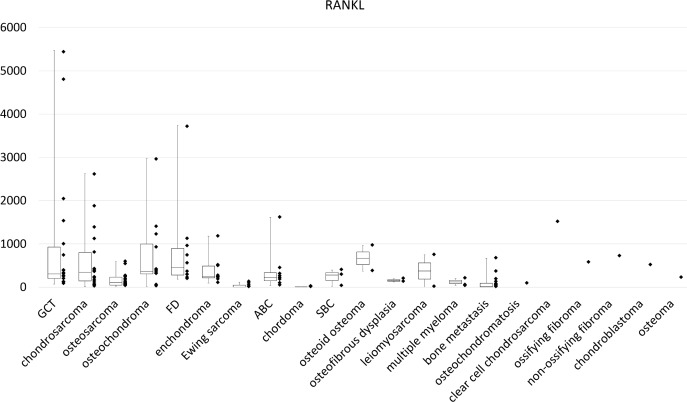
Amplification and dot plot for relative RANKL expression by real-time RT-PCR.

The results of all medians of the experimental value are shown in Table 3. High RANKL expression levels were observed in GCTB, chondrosarcoma, enchondroma, osteochondroma, fibrous dysplasia (FD), and aneurysmal bone cyst (ABC) (Fig 1). The median RANKL expression levels was 346 (range, 74.3–6930) for GCTB, 348 (range, 12.2–2623) for chondrosarcoma, 237 (range, 94.8–1180) for enchondroma, 368 (range, 17.8–2973) for osteochondroma, 452 (range, 186–3737) for FD, 374 (range, 747–1.72) for leiomyosarcoma, and 227 (range, 35.7–1618) for ABC. In histological types with only one sample, RANKL expression level was 1587 for clear cell chondrosarcoma, 718 for non-ossifying fibroma, and 570 for ossifying fibroma. The RANKL expression levels for multiple myeloma and metastatic lesions from solid cancer, for which denosumab is approved, were not very high. The median of RANKL expression level was 45.0 for multiple myeloma and 12.7 for bone metastases, respectively. The post-hoc multiple comparison revealed no statistically significant difference among the histological types.

**Table 3:** The median of RANKL, RANK and OPG expression as assessed with real-time RT-PCR.

Histology	Median		
	RANKL	RANK	OPG
**Giant cell tumor of bone**	346	227	287
**Chondrosarcoma**	348	21.7	2211
**Osteosarcoma**	105	19.3	367
**Osteochondroma**	368	242	1657
**Fibrous dysplasia**	452	89	3731
**Enchondroma**	237	35	1950
**Ewing’ sarcoma**	2.23	2.60	17.4
**Aneurysmal bone cyst**	227	104	358
**Chordoma**	3.41	1.67	2683
**Solitary bone cyst**	283	24.0	367
**Osteoid osteoma**	669	43.8	2988
**Osteofibrous dysplasia**	157	56.6	2915
**Multiple myeloma**	45.0	16.9	370
**Osteochondromatosis**	81	42.7	3693
**Clear cell chondrosarcoma**	1519	43.9	3609
**Ossifying fibroma**	571	26.1	6199
**Non-ossifying fibroma**	718	147	355
**Leiomyosarcoma**	374	44.3	44.9

High levels of RANKL expression were observed for GCTB, chondrosarcoma, enchondroma, osteochondroma, FD and ABC.

### RANK expression

**Fig 2:**
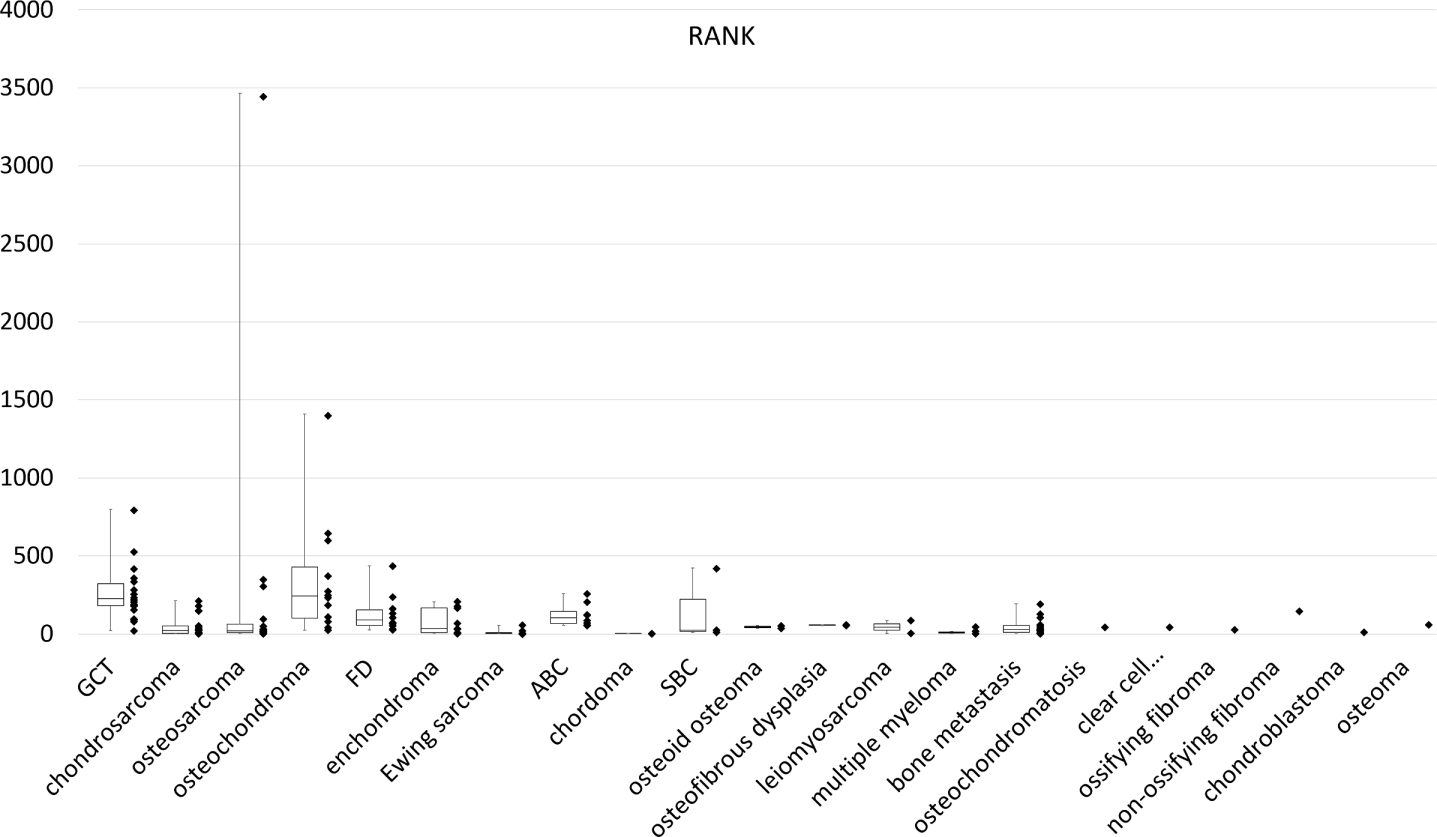
Amplification and dot plot for relative RANK expression by real-time PCR.

The median RANK expression level was 227 (range, 20.9–799) for GCTB, 242 (range, 23.6–1409) for osteochondroma, 104 (range; 55.2–258) for ABC, and 89.1 (range; 26.8–337) for FD (Fig 2). In histological types with only one sample, RANK expression was 147 for non-ossifying fibroma and less than 50 in other histological types. In addition, the median RANK expression in multiple myeloma and bone metastases from solid cancer was 16.9 and 27.9, respectively. The post-hoc multiple comparison revealed no statistically significant difference among the histological types.

### OPG expression

**Fig 3:**
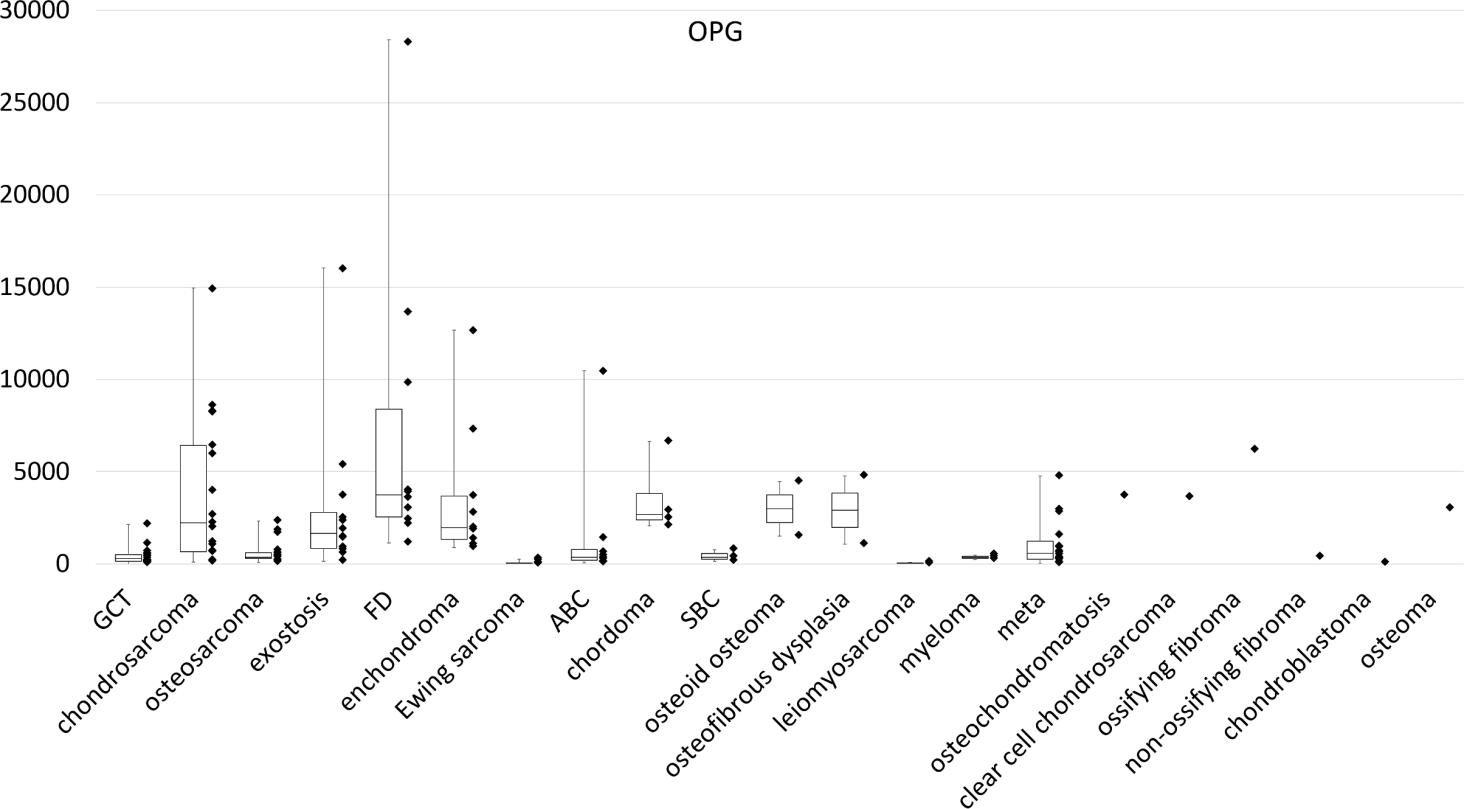
Amplification and dot plot for OPG expression by using real-time PCR.

The median OPG expression level was 3731 (range, 1128–28,413) for FD, 2683 (range, 2069–6643) for chordoma, 2210 (range, 107–14,942) for chondrosarcoma, and 1657 (range, 149–16,049) for osteochondroma (Fig 3). In histological types with only one sample, OPG expression was 6199 for ossifying fibroma, 3693 for osteochondromatosis, and 3609 for clear cell chondrosarcoma. In contrast, the OPG expression level for GCTB was 287 (Fig 3). The post-hoc multiple comparison revealed that expression of OPG in fibrous dysplasia was significantly greater than that of GCT (P = 0.013) and chondrosarcoma (P = 0.032).

### RANKL/OPG ratio

**Fig 4:**
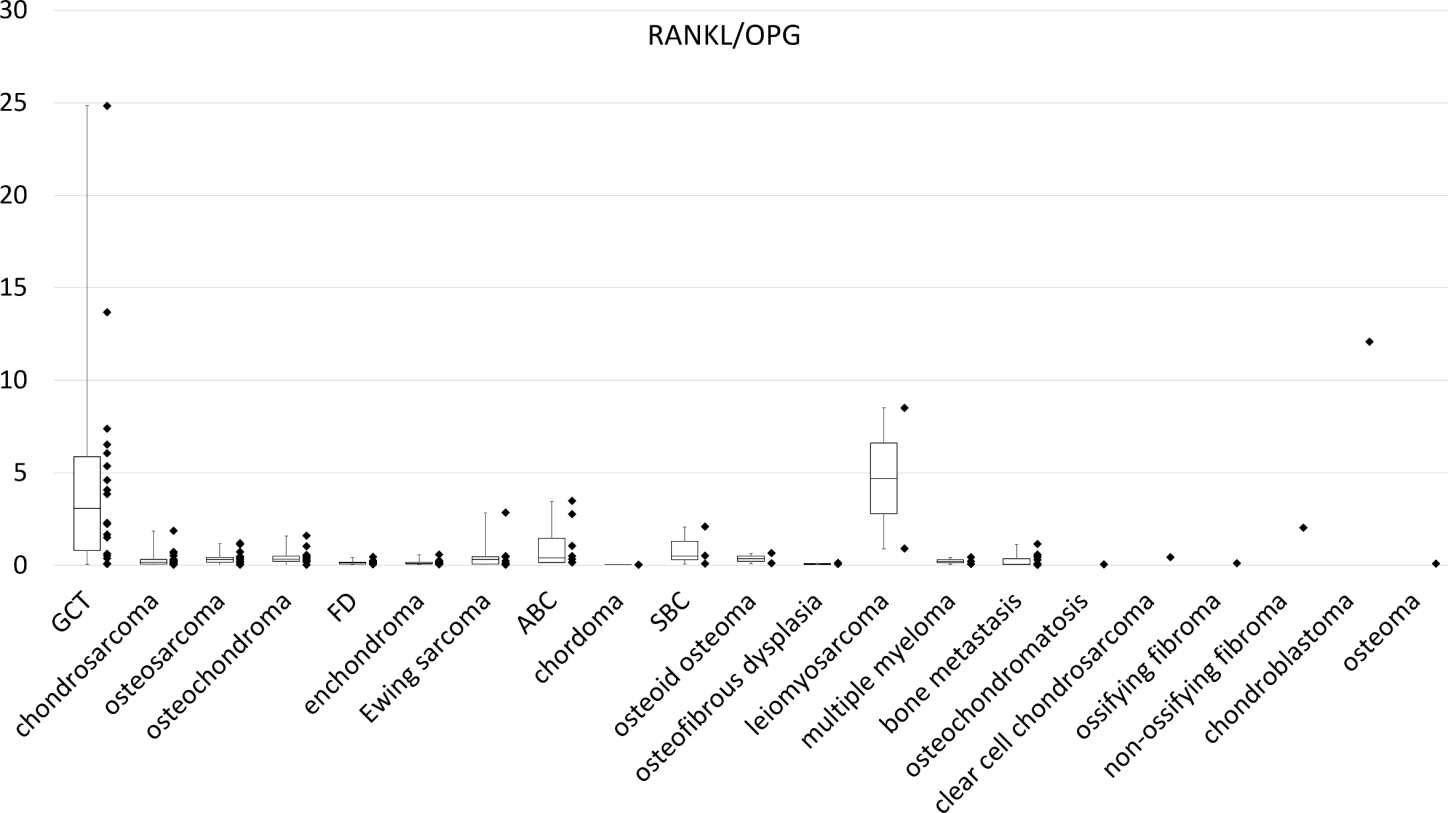
Amplification and dot plot for RANKL/OPG expression by using real-time PCR.

The RANKL/OPG ratio can serve as an index of the osteoclastogenic stimulus [15]. Among all histological types, this ratio was highest in GCTB (3.85), except in chondroblastoma (12.1) and leiomyosarcoma (4.7), which only had one or two samples. The ratio was less than 0.5 in the other histological types (Fig 4). The post-hoc multiple comparison revealed that the RANKL/OPG ratio in GCT was significantly greater than that of chondrosarcoma (P < 0.001), osteosarcoma (P < 0.001), osteochondroma (P = 0.002), fibrous dysplasia (P = 0.002), enchondroma (P = 0.001), Ewing’s sarcoma (P = 0.017) and ABC (P = 0.021).

### Immunohistochemistry

**Fig 5:**
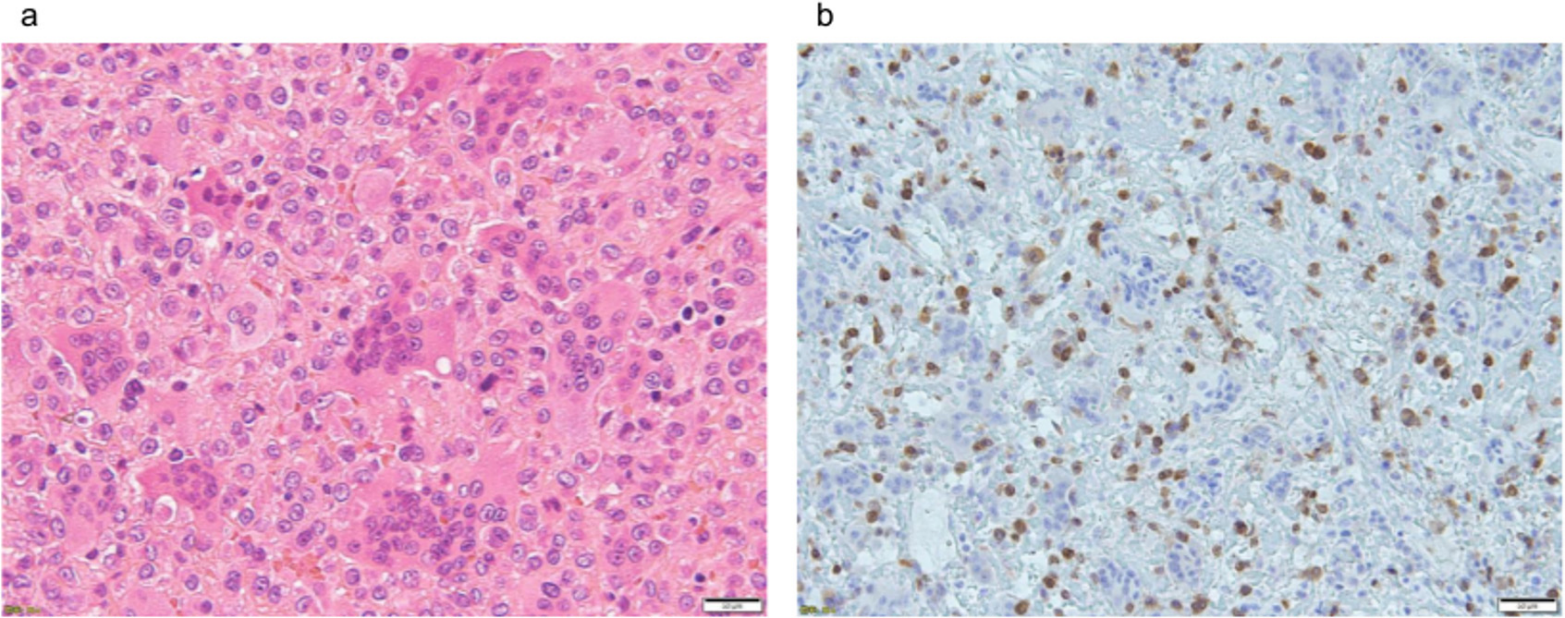
Hematoxylin-eosin and RANKL stains on GCTB. (5a) Tumor stromal cells and osteoclast-like giant cells were observed. (5b) RANKL-positive stromal cells surrounding osteoclast-like giant cells were detected.

**Fig 6:**
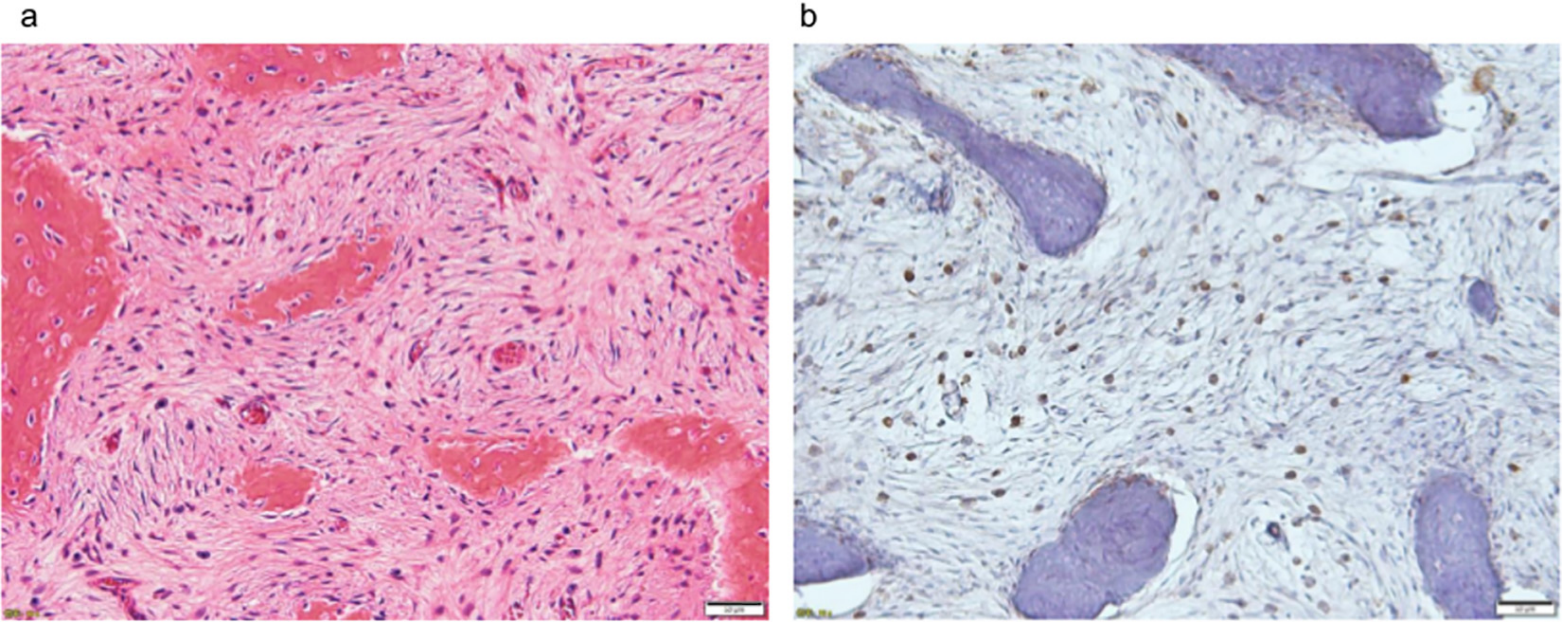
Hematoxylin-eosin and RANKL stains on FD. (6a) Stromal spindle-shaped cells and trabecular of woven bone without osteoblastic rim were observed. (6b) RANKL-positive stromal cells were detected.

**Fig 7:**
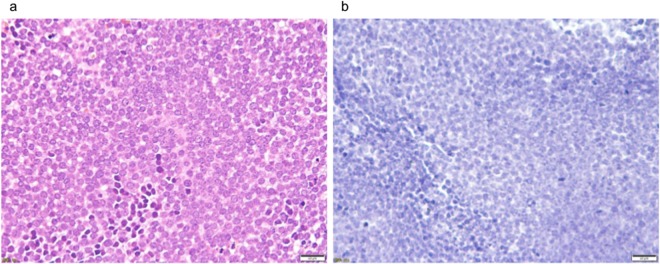
Hematoxylin-eosin and RANKL stains on Ewing’ sarcoma. (7a) Uniform small round cells were observed. (7b) No RANKL-positive cells were detected.

Six RANKL-positive cases were detected: five cases of GCTB and one case of fibrous dysplasia. RANKL-positive stromal cells were detected in five cases of GCTB (Fig 5A and 5B), but not in the other 13 cases of GCTB. None of the five RANKL-positive cases had decalcified tissue sections, and there were no RANKL-positive cells in the 11 cases with decalcified tissue sections. RANKL-positive cells were detected in one fibrous dysplasia case (Fig 6A and 6B). On the other hand, no RANKL-positive cells were detected in multiple myeloma, bone metastases, osteosarcoma, Ewing’ sarcoma, or ABC, which expressed RANKL mRNA (Fig 7A and 7B). RANKL expression could not be assessed in chordoma or chondrosarcoma, which are composed of myxoid parts, because these tissue sections were detached from slides by heat-induced antigen retrieval.

## Discussion

Denosumab is approved as a therapeutic agent for the treatment of multiple myeloma, bone metastases from solid cancer, and GCTB, all of which express RANKL and involve osteoclast activation [4]. After denosumab was approved for the treatment of these tumors, the use of systemic treatment with denosumab as a neoadjuvant therapy was found to successfully reduce the surgical morbidity of tumor resection. Mak et al. reported that biopsy specimens before denosumab showed abundant osteoclast-like giant cells, and operative specimens following denosumab treatment showed severe elimination of osteoclast-like giant cells in GCTB [16]. In particular, GCTB in the spine and recurrent cases of GCTB are considered as good indications for denosumab [17]. RANKL expression has also recently been reported in various other giant cell-rich neoplasms that cause bone resorption. For example, Pelle et al. reported a case of ABC treated with denosumab wherein significant therapeutic effects were noted [11], and histological findings indicated the presence of osteoclast–like giant cells in ABC [14]. In addition, Boyce et al. reported the presence of marked RANKL expression in a biopsy specimen from FD, which was treated with denosumab and demonstrated improvement of pain and tumor growth [12]. In general, all these tumors have shown certain common factors, including the presence of osteoclast-like giant cells and RANKL expression based on immunochemistry. Hence, these tumors were treated as bone tumors with denosumab, despite the off-label use in these histological types of primary bone tumors. Moreover, the reason for the choice of treatment with denosumab in these cases was based on histologic similarities between ABC and GCTB. The presence of osteoclast-like multinucleated giant cells in ABC is similar with the pathogenesis of GCTB, wherein RANKL-mediated bone resorption is essential for tumor growth. Similarly, Akeda et al. reported a case of giant cell reparative granuloma that was successfully treated with denosumab [13].

In the present study, we noted that the relative RANKL expression and the RANKL/OPG ratio of GCTB was highest among all the examined histological types. These values were higher than those for multiple myeloma and bone metastases, for which denosumab is approved. This result indicates a good clinical effect of denosumab for GCTB. Furthermore, the RANKL/OPG ratio appears to be an important index for determining whether denosumab is effective [3]. A case has been reported from our institute wherein a patient with leiomyosarcoma presented a high RANKL/OPG ratio of 8.50. This patient was affected at her left clavicle as a primary lesion, presented with multiple bone metastases, and experienced severe pain. Soon after administration of denosumab for this case, her pain improved, and ossification was noted at the osteolytic lesions on radiography. These findings suggested that a denosumab treatment may possibly be used for bone tumors with a high RANKL/OPG ratio. In contrast, RANKL expression in multiple myeloma and bone metastases from solid cancer (known to express RANKL) was not very high. RANKL expression was higher in chondrosarcoma, osteochondroma, ABC, and FD than in multiple myeloma and bone metastases from solid cancer. Moreover, RANKL protein expression was detected by using immunohistochemistry in ABC and FD, and the efficacy of denosumab administration was reported for these cases. The current results strongly supports these previous reports suggesting denosumab administration for ABC and FD, wherein osteoclast-like giant cells are observed. RANKL expression in chondrosarcoma is as high as that in GCTB, and therefore, it is possible that denosumab may be effective for chondrosarcoma. Moreover, RANKL expression is reported to be high in osteochondroma and enchondroma as well, and hence, cartilage tumors appear to exhibit a relationship with RANKL expression.

Nevertheless, some unclear facts remain concerning the mechanism of bone resorption in primary bone tumor. RANKL expression in chondrosarcoma is high, but the RANKL/OPG ratio is not very high due to the high OPG expression. RANKL expression is high in cartilage tumors such as chondrosarcoma, enchondroma, and osteochondroma, but osteoclasts are not histologically observed in these tumors.

Lee et al. reported that high RANKL expression is related to inferior survival of patients with localized high-grade osteosarcoma, and that RANKL may serve as a promising target for the treatment of osteosarcoma [18]. In the present study, RANKL expression in osteosarcoma was as high as that in multiple myeloma or bone metastatic lesion from solid cancer.

In this study, RANKL mRNA was observed to be higher in some primary bone tumors than in multiple myeloma or bone metastases, both of which are known to express RANKL, although statistical analysis revealed no significant differences in RANKL expression. Furthermore, RANKL/OPG ratio in GCTB was significantly higher than some other bone tumors. These results also support that denosumab is effective for GCTB and that the RANKL/OPG ratio may be a good index in addition to RANKL expression. Although the sample size is too low in some cases to make any comparisons or draw firm conclusions, we think that it is important to investigate RANKL expression in various bone tumors.

RANKL-positive stromal cells were detected by using immunohistochemistry in five cases of GCTB, but not detected in the other 13 cases of GCTB. None of the RANKL-positive cases were decalcified tissue, and no RANKL-positive cell was observed in the 11 cases that were decalcified tissue. We usually prepare decalcified bone tumors and use it for paraffin fixation. Decalcification can reduce antigenicity. However, Branstetter et al. reported that RANKL expression was observed in osteosarcoma sections that had been decalcified [19]. Because of the differences between previous reports and our study, such as the fixation method, antigen retrieval method, primary antibody type, and antigen retrieval buffer, immunohistochemical measurement of the effects of study variables might be affected. We think that the differences are not only due to the decresed antigenicity caused by decalcification but also due to such variables in the staining process.

RANKL-positive cells were not detected in multiple myeloma and bone metastases. These tumors are known to express RANKL, but the level of RANKL protein expression may not be as high as that of RANKL mRNA expression. A RANKL-positive case in fibrous dysplasia was observed from a decalcified tissue section. The RANKL mRNA expression in this case was 3737, which was highest in fibrous dysplasia.

The present findings indicate that some primary bone tumors may offer new therapeutic targets for denosumab, and suggest that denosumab may be effective for tumors expressing RANKL and involving bone resorption by osteoclasts. Previous reports showed the effect of denosumab administration for FD and ABC with systemic symptoms or unresectable lesions that express RANKL [11,12]. Similarly, denosumab may also be effective for patients with aggressive primary bone tumors that are unresectable or exhibit a poor response to preoperative chemotherapy. RANKL is considered to be an important factor in various tumors and can serve as a new promising therapeutic target. Hence, further additional studies are needed for validation.

## Supporting Information

S1 Table All sample data. The values of RANKL, RANK and OPG expression as assessed with real-time RT-PCR in all samples.
(XLSX)
